# Comprehensive Analysis of the Function and Prognostic Value of TAS2Rs Family-Related Genes in Colon Cancer

**DOI:** 10.3390/ijms25136849

**Published:** 2024-06-21

**Authors:** Suzhen Bi, Jie Zhu, Liting Huang, Wanting Feng, Lulu Peng, Liangqi Leng, Yin Wang, Peipei Shan, Weikaixin Kong, Sujie Zhu

**Affiliations:** 1 Institute of Translational Medicine, College of Medicine, Qingdao University, Qingdao 266021, China; bisuzhen2022@163.com (S.B.); huangliting15@126.com (L.H.); fwanting2023@163.com (W.F.); penglulu4541@163.com (L.P.); 15275557898@163.com (L.L.); wangyin@qdu.edu.cn (Y.W.); shanpeipei@qdu.edu.cn (P.S.); 2 Institute for Molecular Medicine Finland (FIMM), HiLIFE, University of Helsinki, 00014 Helsinki, Finland; jie.zhu@helsinki.fi

**Keywords:** colon cancer, type 2 bitter taste receptor, immunotherapy, machine learning

## Abstract

In the realm of colon carcinoma, significant genetic and epigenetic diversity is observed, underscoring the necessity for tailored prognostic features that can guide personalized therapeutic strategies. In this study, we explored the association between the type 2 bitter taste receptor (TAS2Rs) family-related genes and colon cancer using RNA-sequencing and clinical datasets from The Cancer Genome Atlas (TCGA) and the Gene Expression Omnibus (GEO). Our preliminary analysis identified seven TAS2Rs genes associated with survival using univariate Cox regression analysis, all of which were observed to be overexpressed in colon cancer. Subsequently, based on these seven TAS2Rs prognostic genes, two colon cancer molecular subtypes (Cluster A and Cluster B) were defined. These subtypes exhibited distinct prognostic and immune characteristics, with Cluster A characterized by low immune cell infiltration and less favorable outcomes, while Cluster B was associated with high immune cell infiltration and better prognosis. Finally, we developed a robust scoring system using a gradient boosting machine (GBM) approach, integrated with the gene-pairing method, to predict the prognosis of colon cancer patients. This machine learning model could improve our predictive accuracy for colon cancer outcomes, underscoring its value in the precision oncology framework.

## 1. Introduction

Colorectal cancer (CRC) is the second leading cause of cancer-related deaths worldwide [1]. According to the latest global cancer statistics, in 2022, there were 900,000 deaths due to colorectal cancer, accounting for 9.3% of all deaths. Colorectal cancer includes colon cancer and rectal cancer [2]. Colon cancer is a highly heterogeneous malignant tumor, influenced by various factors such as genetics, environment, and diet over the long term [3]. Most colon cancer patients lack obvious symptoms in the early stages, leading to diagnosis in the advanced stages, which poses significant challenges for treatment [4]. Effective biomarkers play a crucial role in early diagnosis of colon cancer patients. However, existing molecular biomarkers, including RAS mutation status, microsatellite instability, and mismatch repair, have significant limitations [5]. Therefore, developing valuable tumor markers and establishing stable prognostic models is crucial for precisely categorizing colon cancer patients into specific risk groups.

In recent years, research has shown significant progress in the use of immunotherapy for the treatment of colon cancer [6]. Highly microsatellite instability (MSI-H)/mismatch repair deficiency (dMMR) is an important biomarker that can predict the effectiveness of immunotherapy for colon cancer. Patients with MSI-H/dMMR colon cancer have a large number of new antigens, enhancing immunogenicity, and are associated with high immune infiltration in the tumor microenvironment (TME) [5]. Immune checkpoint inhibitors such as the PD-1 monoclonal antibodies nivolumab and pembrolizumab, and the CTLA-4 monoclonal antibody ipilimumab have been approved for the treatment of dMMR/MSI-H subtype colon cancer patients [7,8]. However, only 15–20% of colon cancer patients belong to the MSI-H/dMMR subtype, limiting the application of immune checkpoint inhibitors in colon cancer patients [5]. Tumor-infiltrating lymphocytes (TILs) are another biomarker for predicting the response to immunotherapy in colon cancer [9,10]. TILs refer to immune cells infiltrating in tumor tissue, and the type, function, density, and infiltration location of TILs in colon cancer can reflect host defense and tumor progression, with predictive value for patient survival [11]. However, due to the complex relationship between the immune landscape of the TME and the response to tumor immunotherapy, evaluating the response of tumors to immunotherapy through TILs still has limitations. Therefore, finding more predictive biomarkers to stratify patients in the treatment of colon cancer is of great significance.

Colon cancer is mostly a sporadic disease, with diet and lifestyle habits significantly affecting the occurrence and progression of colon cancer [12]. Recent studies have shown a correlation between bitterness sensitivity and cancer development, with individuals highly sensitive to bitterness having an increased risk of cancer [12,13]. Bitter taste sensitivity is mainly associated with the expression and genetic mutations of TAS2Rs [14]. Research in gastrointestinal tumors has revealed that individuals with the TAS2R38 phenylthiourea (PTC) homozygous (AVI/AVI) genotype have a higher risk of developing cancer [15]. Specific gene mutations may make individuals more sensitive to the bitterness of certain compounds, thus increasing the risk of developing specific types of cancer. Singh and colleagues found significant differences in the expression levels of TAS2Rs in different types of breast cancer cell lines, such as decreased mRNA expression levels of TAS2R4 and increased mRNA expression levels of TAS2R14 [16]. However, further research is needed to explore the association between changes in bitter taste receptor genotypes and colon cancer. Current research suggests that bitter substances may impact the development of colon cancer. For instance, the bitter components found in bitter melon are believed to possess anticancer properties, enhancing the inhibitory effects of doxorubicin on the proliferation of colon cancer cells [17]. TAS2Rs are widely expressed in the intestines and are involved in regulating physiological processes such as food intake, gut microbiota balance, and cancer development [18]. Therefore, bitter substances may affect the occurrence and progression of colon cancer by activating or regulating the expression of TAS2Rs.

TAS2Rs are a type of chemosensory G protein-coupled receptors, with a total of 25 functional gene-encoded bitter taste receptor proteins (TAS2R1, 3, 4, 5, 7, 8, 9, 10, 13, 14, 16, 19, 20, 30, 31, 38, 39, 40, 41, 42, 43, 45, 46, 50, 60) found in human cells [14]. These receptors are not only expressed in the oral cavity but also exist in the gastrointestinal tract and other extrabuccal tissues [19,20]. Although less is known about the extrabuccal functions of TAS2Rs, some evidence suggests that TAS2Rs may represent a receptor system utilized by different cell types to perceive external stimuli, including immune cells [21] and tumor cells. TAS2Rs are expressed on various immune cells, including lymphocytes and myeloid cells [22]. The most expressed bitter taste receptors in lymphocytes are TAS2R10 and TAS2R38, especially in CD4+ T lymphocytes [23]. In macrophages, transcripts of at least 16 different subtypes of bitter taste receptors have been detected. Bitter taste agonists inhibit cytokine release and cell migration in resident macrophages and macrophages derived from monocytes, and stimulate the release of reactive substances [24,25]. Furthermore, the increased risk of cancer development is associated with various bitter taste receptor genes [26,27], and the functional response depends on the activated cell type. The activity of bitter taste receptors may stimulate intestinal motility to promote gastric acid secretion, stimulate enteroendocrine cells to secrete pancreatic glucagon, and reduce lipopolysaccharide-mediated release of interleukin-6 (IL-6) from white blood cells, thus affecting cancer development. Currently, the role of TAS2Rs in carcinogenesis and their function in therapy remains incompletely understood, but their importance in cancer development and immune regulation has begun to receive widespread attention from researchers.

Machine learning models are increasingly being used in the prognosis of various diseases due to their ability to analyze large datasets and identify complex patterns that may not be apparent through traditional statistical methods [28,29]. These machine learning models offer several advantages over traditional statistical models such as the Cox proportional hazards model, particularly in capturing nonlinear relationships, handling high-dimensional data, and exhibiting robustness to missing data [30,31]. In this study, together with the Cox model, we utilized three types of machine learning models to enhance the predictive accuracy of colon cancer prognosis.

In conclusion, we propose a hypothesis that bitter substances may influence the immune cell infiltration of colon cancer patients through bitter taste receptors, thereby affecting the survival of colon cancer patients. In this study, we aim to investigate the role of TAS2Rs in colon cancer by analyzing colon cancer gene expression and survival data from the TCGA and GEO databases. Based on seven TAS2Rs genes associated with prognosis, we constructed two molecular subtypes of colon cancer and analyzed the survival, immune cell infiltration, and biological processes of each colon cancer subtype. Furthermore, we developed a gradient boosting machine (GBM) scoring model that demonstrated stability in an internal test set and three external test sets compared with Cox, random forest (RF), and conditional inference forest (CIF) models. Furthermore, based on this GBM model, we also constructed a nomogram containing various clinical information including age and stage, which exhibited strong predictive accuracy regarding the risk assessment of colon cancer patients, with an ROC value of more than 0.65 at 1, 2, and 3 years in three external test sets.

## 2. Results

### 2.1. Differential Expression and Prognostic Value Analysis of TAS2Rs Gene Family in Colon Cancer

**Figure 1:**
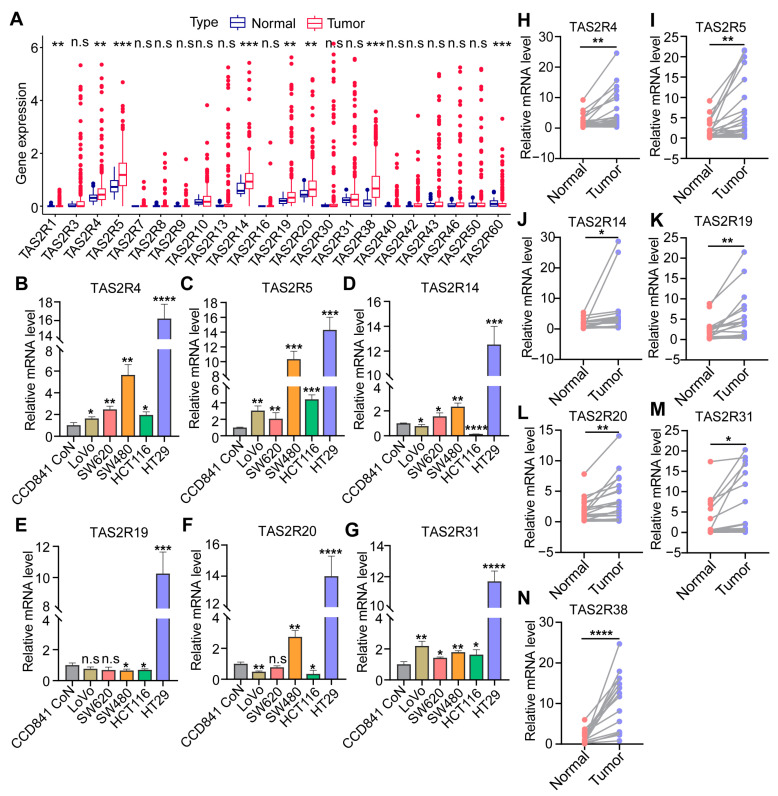
Expression of the TAS2Rs gene family in colon cancer tissues and cell lines. (**A**) Expression of 25 TAS2Rs gene family members in normal colon tissues and colon cancer tissues in TCGA-CDAD. (**B**–**G**) Expression of TAS2R4, TAS2R5, TAS2R14, TAS2R19, TAS2R20, and TAS2R31 in human normal colonic epithelial cell lines and different types of colon cancer cell lines. (**H**–**N**) Expression of TAS2R4, TAS2R5, TAS2R14, TAS2R19, TAS2R20, TAS2R31, and TAS2R38 in colon cancer tissues compared to normal colon tissues. The upper and lower ends of these boxes represent the interquartile range of the values. The line inside the box represents the median. Asterisks indicate statistical significance (* *p* < 0.05, ** *p* < 0.01, *** *p* < 0.001, **** *p* < 0.0001).

Currently, 25 TAS2Rs genes have been identified in human somatic cells, which are not only present in the mouth but also expressed in extralingual tissues such as the upper and lower respiratory tract, intestines, adipose tissue, brain, heart, and immune cells, participating in various physiological processes [14,32]. Utilizing univariate Cox regression analysis, we identified seven TAS2Rs genes associated with prognosis in colon cancer patients, including TAS2R4, TAS2R5, TAS2R14, TAS2R19, TAS2R20, TAS2R31, and TAS2R38 (Supplementary Figure S2). Differential expression analysis results revealed that compared to normal colon tissue, colon cancer samples exhibited elevated expression levels of TAS2R1, TAS2R4, TAS2R5, TAS2R14, TAS2R19, TAS2R20, and TAS2R38 (*p* < 0.05), with the exception of TAS2R60, which was downregulated (Figure 1A). After further research, we conducted a detailed analysis of the expression of seven TAS2Rs genes in human normal colonic epithelial cell line CCD841 and five colon cancer cell lines LOVO, SW620, SW480, HCT116, and HT29. The study results revealed significantly higher expression levels of these seven TAS2Rs genes in cancer cells compared to normal colonic epithelial cells, particularly noted in the HT29 cell line (Figure 1B–G, *p* < 0.05). Here, we analyzed the genetic mutation status of five colon cancer cell lines. HT29 cells stand out from the others by not having the KRAS mutation and containing the BRAF mutation (Supplementary Table S1). However, we examined the expression of TAS2Rs genes in the presence of KRAS and BRAF mutations, or non-mutation states, and observed differences in the expression of these genes between the mutation and nonmutation groups (Supplementary Figure S3). Similarly, analysis of clinical colon cancer tissue samples revealed that the expression levels of the seven prognostic TAS2Rs genes are also elevated in colon cancer tissue samples compared to normal colon tissue (Figure 1H–N, *p* < 0.05). We evaluated the protein expression levels of TAS2R38 in colon cancer tissues and normal colon tissues using the immunohistochemistry staining data from the HPA database (http://www.proteinatlas.org/, accessed on 6 June 2024). The results indicate a significantly elevated expression level of TAS2R38 in colon cancer tissues (Supplementary Figure S4). These results indicate a close correlation between the expression levels of these TAS2Rs genes and the survival rate of colon cancer patients, further emphasizing the importance of bitter taste receptors in colon cancer.

### 2.2. Constructing Colon Cancer Molecular Subtypes Based on Seven TAS2Rs Prognostic Genes

**Figure 2:**
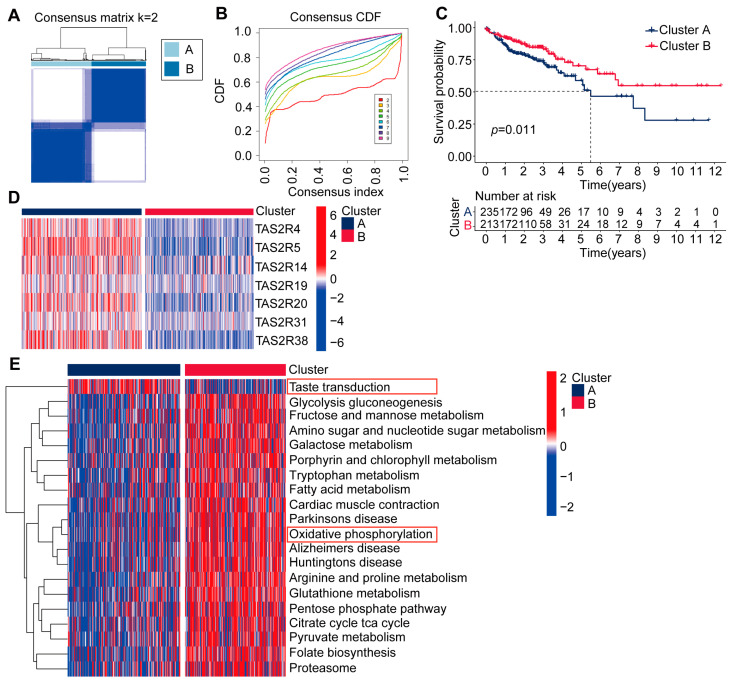
Identification and biological characteristics of two molecular subtypes of colon cancer. (**A**) Consensus matrix of TCGA-COAD for k = 2. (**B**) CDF curves in consensus clustering analysis. CDF curves representing consensus scores for different subtype numbers (k = 2–9). (**C**) Survival curves of TCGA-COAD patients among different subtypes. Survival rate differences were evaluated using log-rank test, *p* < 0.05. (**D**) Heatmap of seven TAS2Rs prognostic genes in the TCGA-COAD dataset. (**E**) GSEA enrichment analysis showing the activation status of biological pathways in two molecular subtypes of colon cancer, red boxs indicates an important signaling pathway.

Based on the seven TAS2Rs prognostic genes, we performed unsupervised clustering analysis on colon cancer patients using the R package ConsensusClusterPlus, identifying two molecular subtypes, namely Cluster A and Cluster B, consisting of 235 and 213 patients, respectively (Figure 2A–C). Survival analysis results indicated that colon cancer patients in Cluster B had a significant survival advantage compared to those in Cluster A (Figure 2C). Further research has revealed that the expression trends of the seven TAS2Rs genes differ in two molecular subtypes of colon cancer. Specifically, in Cluster B with higher survival rates, the expression levels of these seven TAS2Rs genes are lower, while in Cluster A, with poorer survival rates, the expression levels of these seven TAS2Rs genes are higher (Figure 2D). These findings are consistent with the differential analysis results (Figure 1), suggesting that potential adverse impact of elevated TAS2Rs gene expression on colon cancer prognosis. To explore the biological differences between these two colon cancer molecular subtypes, we used the GSVA method to analyze the biological functions of the two subtypes. The analysis results indicate that Cluster A is associated with the taste transduction signaling pathway, while Cluster B is related to signaling pathways such as fatty acid metabolism, oxidative phosphorylation, and glucose metabolism (Figure 2E). This result suggests that, compared to Cluster A, Cluster B may have a survival advantage in biological processes such as fatty acid metabolism, oxidative phosphorylation, and glucose metabolism due to the lower expression levels of TAS2Rs genes.

Research reports indicate that there are differences in the incidence of cancer among different ethnicities. For example, Black individuals are more prone to prostate cancer, White individuals are more susceptible to lung cancer, and Asians are more likely to develop liver cancer [33]. Additionally, studies have found that the occurrence and progression of colon cancer are also influenced by racial differences [34]. Based on this discovery, it is speculated that the expression of TAS2Rs may vary among different races. We conducted a study analyzing the expression of seven TAS2Rs genes in White, Black or African American, and Asians using the TCGA-COAD dataset. The results showed that the expression levels of these seven TAS2Rs genes in White individuals were significantly higher compared to Black or African American, and Asian individuals, especially the TAS2R4, TAS2R14, TAS2R19, and TAS2R31 genes (Supplementary Figure S5A). We also analyzed the proportions of White, Black or African American, and Asian individuals in the two subtypes and found no differences among these three races in the two subtypes (Supplementary Figure S5B). Further analysis indicated that regardless of race, Cluster B had a better survival rate than Cluster A (Supplementary Figure S6A–C), and was particularly more pronounced in White individuals (Supplementary Figure S6A), possibly due to the higher expression of TAS2Rs genes in White individuals. Gender is also a significant factor leading to survival differences in colon cancer patients [35]. We analyzed the expression of the seven TAS2Rs genes in males and females, and the results showed no significant difference in gene expression between genders (Supplementary Figure S5B). Both subtypes also show no difference in the proportions between genders (Supplementary Figure S5D). However, an analysis of the survival status of the two subtypes between different genders revealed that the survival difference in males is more significant (Supplementary Figure S6D,E), indicating indeed a difference in survival among colon cancer patients of different genders. In conclusion, racial and gender differences impact the progression of colon cancer.

The development of colon cancer involves various genetic and epigenetic changes. Studies have shown that genetic alterations in TAS2Rs gene expression may lead to an increase in bitter taste sensitivity [36]. Therefore, it can be speculated that TAS2Rs genotyping is related to epigenetics. We analyzed the expression of RNA methylation (m6A and m5C) and DNA methylation (5mC) regulatory factors in the two molecular subtypes of colon cancer. The results revealed that in the low-expressed Cluster B of TAS2Rs genes, the expression levels of m6A regulatory factors METTL3, METTL14, RBM15, ZC3H13, FTO, YTHDC1, YTHDC2, YTHDF1, YTHDF3, IGF22BP1, HNRNPA2B1, FMR1, and LRPPRC were lower, and we also found that ALKBH5 had higher expression levels in Cluster B (Supplementary Figure S7A). Consistent with previous research findings, CBLL1, FTO, LRPPRL, METTL3, RBM15, YTHDF1, and ZC3H13 are expressed at high levels in colon cancer tissues, while their expression levels are low in normal colon tissues; ALKBH5 is underexpressed in colon cancer tissues but overexpressed in normal colon tissues [37]. In addition, the m5C methylation regulatory factors NSUN3, NSUN6, and NSUN7 exhibit low expression levels in Cluster B, whereas ALYREF and YBX1 show high expression levels in Cluster B (Supplementary Figure S7B). Research has shown that the 5mC regulatory factor DNMT3A plays a crucial role in colitis-associated cancer, with low expression being favorable for patient prognosis [38]. Our research also found that DNMT3A is expressed at lower levels in Cluster B with low expression of TAS2Rs genes. Furthermore, regulatory factors of 5mC such as DNMT3B, TET2, TET3, MBD4, MECP2, NEIL1, and ZBTB4 show lower expression levels in Cluster B (Supplementary Figure S7C). These results suggest that RNA methylation and DNA methylation may play important roles in colon cancer, with the two molecular subtypes of colon cancer being associated with different methylation patterns, providing crucial clues for further research into the pathogenesis and treatment of colon cancer.

### 2.3. Immune Characteristics of Two Molecular Subtypes of Colon Cancer

**Figure 3:**
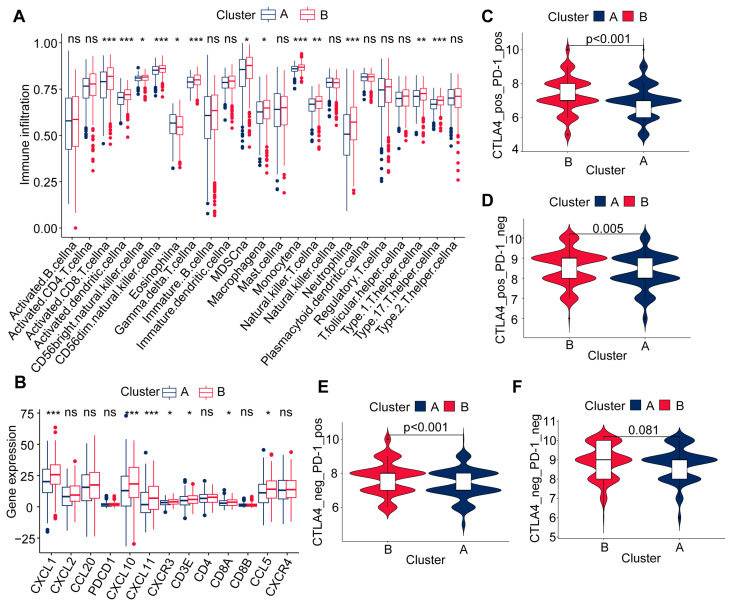
Immune cell infiltration characteristics and immune therapy prediction in the two molecular subtypes of colon cancer. (**A**) Analysis of immune cell content in the TCGA-COAD dataset. (**B**) Analysis of differential expression of chemokines in the TCGA-COAD dataset. Intergroup comparisons were performed using one-way analysis of variance (* *p* < 0.05, ** *p* < 0.01, *** *p* < 0.001). (**C**–**F**) Relationship between immune therapy-related scores and patient subtypes in the TCGA-COAD dataset. The Wilcoxon test was used to conduct the intergroup comparisons between the different subtypes.

TME refers to the environment surrounding tumor cells, including immune cells, vascular cells, extracellular matrix, and other components [39]. These components interact with tumor cells, significantly influencing the growth, spread, and response to treatment of tumors. Immune infiltration refers to the process of immune cells entering the tumor microenvironment. These immune cells, including but not limited to T cells, natural killer cells (NK cells), and macrophages, play a crucial role in combating tumors. Depending on the degree of immune cell infiltration, tumors can be classified as “cold” tumors with low immune cell infiltration and “hot” tumors with high immune cell infiltration [40]. In this study, we compared the immune cell composition of the two molecular subtypes of colon cancer and found significant differences in the TME cell-type composition (Figure 3A). This difference indicates significant variations in immune therapy response among different subtypes of colon cancer. Particularly, Cluster B is rich in various immune cells with tumor-killing effects, such as CD8+ T cells, NK cells, natural killer T cells, as well as antigen-presenting macrophages and dendritic cells (Figure 3A). CD8+T cells release granzymes and perforin to lyse the target cancer cells [41]. We analyzed the expression of the effector molecules of cytotoxic CD8+ T lymphocytes (CTLs) Granzyme B (GZMB), and Perforin (PRF1) encoding genes in the two subtypes of colon cancer. The results indicated that there was no difference in the expression levels of GZMB between Cluster A and Cluster B, while the expression of the PRF1 gene was higher in Cluster B and lower in Cluster A (Supplementary Figure S8A). Additionally, we analyzed the expression of MHC-I molecules on cell surfaces [42] that can be correctly recognized by CD8+ T cells, revealing higher immune infiltration of MHC-I molecules in Cluster B compared to Cluster A (Supplementary Figure S8B). The ability of CD4+ T cells independent of CD8+ T cells in participating in antitumor immunity is increasingly recognized. Research has found that Th1-type cell-directed CD4+ T cells reprogram bone marrow cells of the tumor to express interferon-activated antigen presentation and iNOS-mediated tumor-killing phenotype, thereby inducing the immune-escape tumor to undergo inflammatory cell death [43]. Our research reveals that the gene encoding iNOS (NOS2) is highly expressed in Cluster B (Supplementary Figure S8A) and shows a strong correlation with immune function (antigen presentation and interferon response) pathways in Cluster B (Supplementary Figure S8B). Additionally, we analyzed the expression of macrophage M1 (CD86, CD80) and M2 (CD206, CD163) markers in the two subtypes of colon cancer and found no difference in the expression of M1 and M2 markers between Cluster A and Cluster B (Supplementary Figure S8C). These results suggest that patients in Cluster B may benefit more from immunotherapy.

Furthermore, our evaluation of the differential expression of chemokines and their receptors revealed that Cluster B, which showed a positive response to immune therapy, exhibited elevated expression levels of the chemokines CXCR3 and CXCL10. In contrast, Cluster A displays diminished expression levels of the antitumor chemokines CXCL8 and CXCL10 (Figure 3B), suggesting a reduced effectiveness in immune therapy. To validate this finding, we performed an in-depth analysis using immune data from colon cancer patients in the TICA database. The results showed that patients in Cluster B had a better response to PD-1/CTLA4, while patients from Cluster A had a poorer response (Figure 3C–F). This research discovery provides new insights into immune therapy for colon cancer.

### 2.4. Generation and Function of DEGs in Two Molecular Subtypes of Colon Cancer

**Figure 4:**
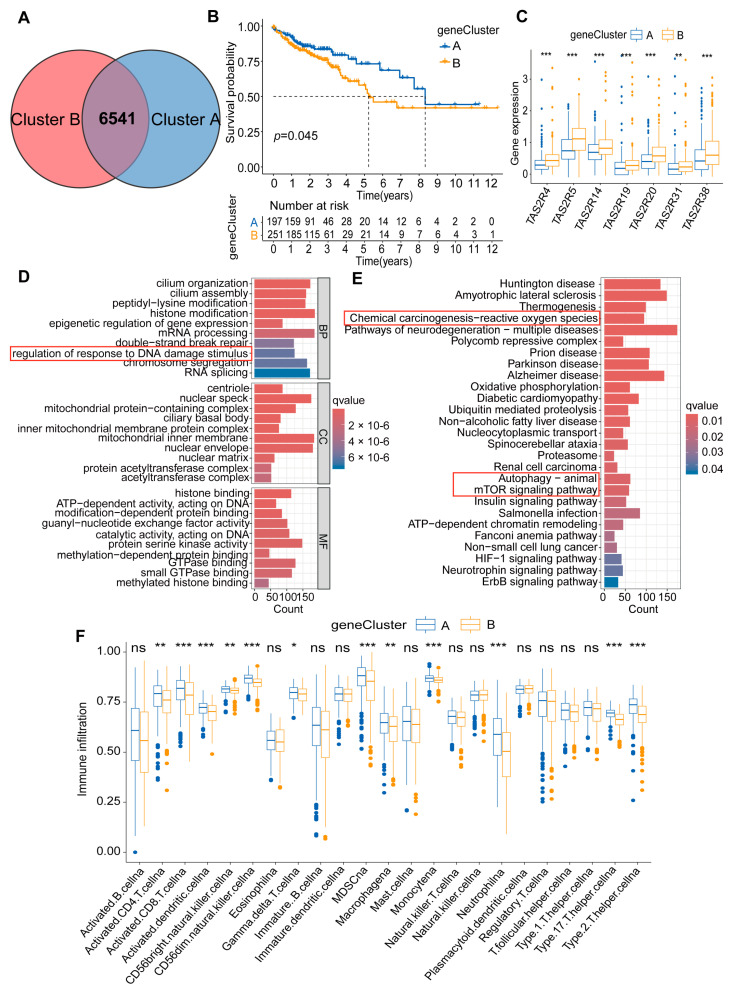
Construction and biological processes in colon cancer genomic subtypes. (**A**) Venn diagram of 6541 TAS2Rs phenotype genes. (**B**) Survival curve of patients with colon cancer between TAS2Rs genomic subtypes geneCluster A and geneCluster B; 197 patients belong to geneCluster A and 251 patients belong to geneCluster B (*p* < 0.05). (**C**) Expression of the seven TAS2Rs prognostic genes in the two genome subtype clusters. (**D**) Functional annotation of TAS2Rs phenotypic genes using GO enrichment analysis, red boxs indicates an important signaling pathway. (**E**) Enrichment analysis of signal pathways for TAS2Rs phenotypic genes using KEGG enrichment analysis, red boxs indicates an important signaling pathway. (**F**) Abundance of immune cells in the two genome subtype clusters (* *p* < 0.05, ** *p* < 0.01, *** *p* < 0.001).

To investigate the potential biological behaviors of colon cancer molecular subtype, we successfully identified 6541 colon cancer-related DEGs using the R package limma (Figure 4A). Firstly, employing unsupervised clustering, we classified colon cancer patients into two genomic subtypes based on the expression patterns of TAS2Rs phenotype genes, named geneCluster A and geneCluster B (Figure 4B; Supplementary Figure S9). We observed that patients in these two clusters exhibited opposite prognostic patterns; notably, patients in geneCluster A exhibited more favorable prognosis compared to those in geneCluster B (Figure 4B). Further analysis revealed that geneCluster B was characterized by heightened expression of the seven TAS2Rs prognostic genes, while geneCluster A displayed reduced expression levels of these genes (Figure 4C). This aligned with previous findings indicating an association between a high expression of TAS2Rs in colon cancer patients and poor survival rates (Figure 2C,D).

This extensive set of DEGs serves as a vital resource for exploring the molecular mechanisms of colon cancer. Subsequently, GO enrichment analysis showed that these DEGs were enriched in biological processes related to the regulation of DNA damage response and mismatch repair (Figure 4D). Additionally, KEGG enrichment analysis linked these genes to critical features, including chemical carcinogenesis of reactive oxygen species, autophagy, and the mTOR signaling pathway (Figure 4E), further revealing key molecular events in the development of colon cancer. Additionally, analysis of the composition of immune cells in the two clusters revealed different patterns of immune cell infiltration. Specifically, geneCluster B showed lower levels of immune cells (including CD4+ T cells, CD8+ T cells, dendritic cells, NK cells, macrophages, etc.), while geneCluster A exhibited higher levels of these immune cells (Figure 4F). This finding suggests that TAS2Rs-related genes play an important role in regulating immune cell infiltration in different TMEs. These findings provide important clues for further exploring the molecular mechanisms of colon cancer and developing new treatment strategies.

### 2.5. Construction and Validation of the Scoring Model Based on Two Molecular Subtypes of Colon Cancer

**Figure 5:**
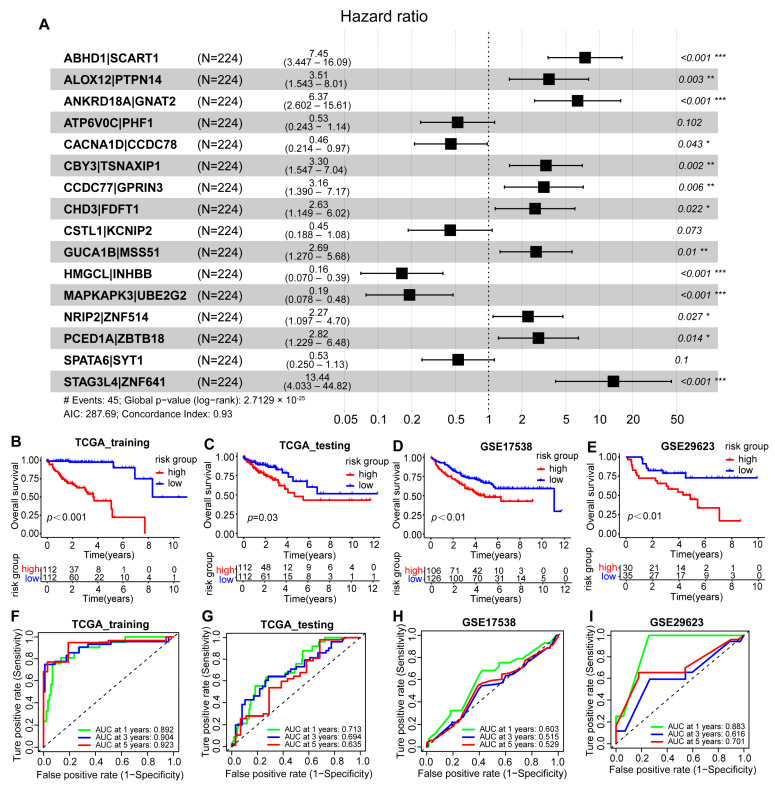
Construction of the GBM scoring model based on gradient boosting machine (GBM) learning. (**A**) Coefficients of the 16 gene pairs used to build the GBM scoring model (* *p* < 0.05, ** *p* < 0.01, *** *p* < 0.001). (**B**–**E**) Survival curves of the GBM scoring model in the TCGA-COAD training set, TCGA-COAD test set, GSE17538, and GSE29623 (log-rank test). (**F**–**I**) ROC curves for 1, 3, and 5 years GBM scoring models in the TCGA-COAD training set, TCGA-COAD test set, GSE17538, and GSE29623.

Currently, the development of robust prognostic models is crucial for enhancing disease treatment outcomes and the patient quality of life. By pairing 381 phenotype genes of two molecular subtypes of colon cancer, we obtained 381×380/2 (72,390) gene pairs. Among these gene pairs, we identified over 18,000 pairs where the frequency of “Gene A > Gene B” falls between 20% and 80%, indicating that these gene pairs carry sufficient information to predict survival status. Subsequently, we utilized univariate Cox regression analysis and LASSO regression analysis (Supplementary Figure S10) to select 30 gene pairs with statistical significance from numerous gene pairs for building the prognostic model. As shown in Figure 5A, multivariate Cox regression analysis identified 16 gene pairs significantly associated with survival differences, including 10 high-risk gene pairs (HR > 1, *p* < 0.05) and 6 low-risk gene pairs (HR < 1, *p* < 0.05). The selection of these gene pairs was based on their significant relevance in predicting the survival status of colon cancer patients. Using these 16 gene pairs, we applied four machine learning methods to construct the prognostic models, among which the model built on the GBM machine learning method demonstrated better performance, as detailed below:

Sum = 7.448 × ABHD1|SCART1 + 3.515 × ALOX12|PTPN14 + 6.374 × ANKRD18A|GNAT2 + 0.526 × ATP6V0C|PHF1 + 0.457 × CACNA1D|CCDC78 + 3.300 × CBY3|TSNAXIP1 + 3.158 × CCDC77|GPRIN3 + 2.630 × CHD3|FDFT1 + 0.450 × CSTL1|KCNIP2 + 2.686 × GUCA1B|MSS51 + 0.165 × HMGCL|IHBB + 0.193 × MAKAPK3|UBE2G2 + 2.270 × NRIP2|ZNF514 + 2.822 × PCED1A|ZBTB18 + 0.531 × SPATA6|SYT1 + 13.444 × STAG3L4|ZNF641

GBM score = e^Sum^

To validate the prognostic performance of the GBM scoring model, we conducted tests in three colon cancer cohorts, including two external test sets and one internal test set. By using the median value of each cohort as the threshold to divide into high-risk and low-risk groups, we found that despite disease heterogeneity and intercohort differences, the GBM-based scoring model had better performance (*p* < 0.05, log-rank test) compared to other methods including Cox, RF, and CIF. Survival analysis results indicated that patients in the high-risk group had a poorer prognosis across all cohorts, while those in the low-risk group had a better prognosis (Figure 5B–E, *p* < 0.05). In the training set, the AUC-ROC values for 1, 3, and 5 years were all above 0.8 (Figure 5F), while in the other three test sets, the AUC-ROC values for 1, 3, and 5 years are all above 0.5 (Figure 5G–I). This finding highlights the strong predictive ability and potential clinical utility of the GBM scoring model in predicting the prognosis of colon cancer patients. Although the prognostic models built on the three other machine learning methods performed well in the TCGA-COAD training set, with AUC-ROC values greater than 0.9 at 1, 3, and 5 years, these models showed poorer prognosis for the high-risk group and better prognosis for the low-risk group. However, the results in the other three validation sets were not ideal (Supplementary Figures S11–S13). This comparison further demonstrates the superiority of the GBM model in handling complex biological data and avoiding batch effects, making it the ultimate choice for a predictive model.

### 2.6. Construction and Verification of the GBM Scoring Model Nomogram

**Figure 6:**
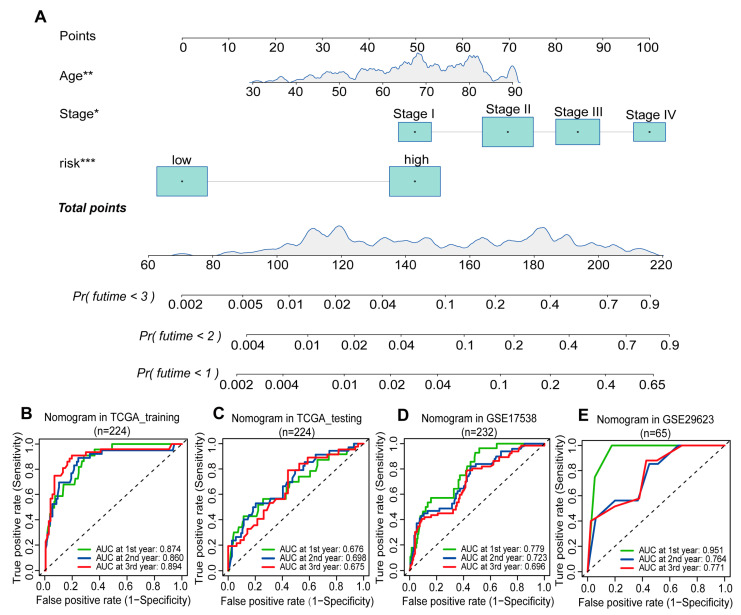
Generation and verification of the nomogram. (**A**) A three-factor nomogram with high and low risk scores (* *p* < 0.05, ** *p* < 0.01, *** *p* < 0.001). (**B**) ROC curve of the TCGA-COAD training set. (**C**) ROC curve of the TCGA-COAD test set. (**D**) ROC curve of GSE17538. (**E**) ROC curve of GSE29623.

For the ease of use of the GBM scoring model, we created a nomogram containing assorted clinical information. The TCGA-COAD dataset was randomly divided into a training set (*n* = 224) and a test set (*n* = 224). In the training set, we conducted multivariable independent prognostic analysis based on high- and low-risk scores and other clinical information (such as age, staging). After identifying independent risk factors, we further established a nomogram depicting the relationships between these factors within the training set (Figure 6A). To assess the effectiveness of the nomogram, we predicted patient survival status at 1, 2, and 3 years and plotted the corresponding ROC and calibration curves (Figure 6B–E; Supplementary Figure S14). We found that the average AUC-ROC was greater than 0.8, indicating good classification performance of the nomogram (Figure 6B). We compared the AUC-ROC values of the training set with the external validation sets using DeLong’s test [44]. Our analysis revealed a significant difference between the training set and the GSE17538 set (*p*-value < 0.05). However, there was no significant difference between the training set and the GSE29623 set (*p*-value > 0.05) (Supplementary Figure S15). Despite some signs of overfitting in the training set based solely on the ROC curve, considering the calibration curve revealed high accuracy in both the training and test sets, demonstrating the robustness of the nomogram (Supplementary Figure S14A,B). To further validate the model’s generalizability, we analyzed the survival status of colon cancer patients at 1, 2, and 3 years using the validation set data from GSE17538 and GSE29623 (Figure 6D,E; Supplementary Figure S14C,D). The results from these external test sets were consistent with the training set, further confirming the effectiveness and robustness of the model.

## 3. Discussion

High heterogeneity at the genetic and gene regulation levels leads to differences in the treatment response of colon cancer, resulting in variations in colon cancer survival rates [45]. Traditional prognostic approaches for colon cancer mainly rely on the TNM staging system [46], histopathological standards [47], molecular markers [48], and tumor cell differentiation [49]. While these indicators can somewhat assist doctors in assessing patients’ prognoses, they often fall short in accurately identifying high-risk individuals who could benefit from personalized treatments. Therefore, to enhance the survival rates of colon cancer patients, there is an urgent need to identify more precise biomarkers and select appropriate immunotherapies. In our current research, we have identified two new molecular subtypes of colon cancer based on TAS2Rs family-related genes associated with patient prognosis. These subtypes not only exhibit molecular uniqueness but also hold significant prognostic value for the survival of colon cancer patients. Additionally, we developed a GBM scoring model based on gene-pairing methods. The model categorizes colon cancer patients into different risk groups based on their scores. Based on this model and the clinical information from colon cancer patients, we also created a nomogram, aiming to assist doctors and patients in better understanding the severity of the disease and the urgency of treatment.

TAS2Rs are biological markers for bitter taste reactions in the human body, and variations in their genotypes may affect an individual’s sensitivity to bitterness, thus influencing dietary habits and nutritional intake, factors that have been linked to cancer risk [27,50]. This article aims to explore the relationship between TAS2Rs gene expression and the risk of cancer occurrence. Studies by Singh et al. indicate that the expression level of TAS2R4 is lower in breast cancer cell lines (MCF-7 and MDA-MB-231) compared to noncancerous breast epithelial cell lines (MCF-10A) [16]. In contrast, the expression level of TAS2R14 in breast cancer cell lines is higher than in the noncancerous control group, suggesting that TAS2R14 may have different mechanisms of action in breast cancer development [51]. These findings reveal diverse expression patterns of TAS2Rs genes in different types of cancer, implying their complex roles in cancer development. Our results show that compared to normal colon tissue, the expression levels of multiple bitter taste receptor genes are elevated in colon cancer tissue, including TAS2R4, TAS2R5, TAS2R14, TAS2R19, TAS2R20, TAS2R31, and TAS2R38. This suggests that bitter taste receptor genes may play a promoting role in the development of colon cancer. Importantly, through analysis of colon cancer patients, researchers found a negative correlation between high expression of TAS2Rs genes and the quality of patient prognosis, indicating that patients with high levels of TAS2Rs gene expression have a poorer prognosis. Furthermore, based on the discovery of two new molecular subtypes, the research further reveals the TME characteristics under different TAS2Rs gene expression patterns. Among them, the characteristics of Cluster A subtype are “cold” tumors with less immune cell infiltration, while Cluster B subtype consists of “hot” tumors with abundant immune cell infiltration. Notably, colon cancer patients belonging to Cluster B show a higher response rate to immunotherapy, suggesting that TAS2Rs gene expression patterns may serve as new biomarkers for predicting tumor response to immunotherapy.

Today, machine learning methods are used in a wide range of oncology-related diagnoses, including colon polyp detection, breast cancer X-ray screening, and glioma classification [52]. Some studies have shown that on the basis of the combination of biomarkers and multiple clinical factors, the prediction accuracy of traditional statistical methods based on Cox regression and logistic regression is higher [53,54]. However, traditional statistical methods commonly used by clinicians overlook the more complex nonlinear interactions between variables, leading to poor generalization and model calibration, which limits their utility in clinical decision support [52,55]. In contrast to traditional regression-based methods, gradient boosting machine (GBM) is a decision tree-based machine learning model capable of handling high-dimensional data and nonlinear relationships [54]. In this context, we proposed a GBM scoring model based on a gene-pairing method. By analyzing the differences in paired gene expressions, we effectively reduce batch effects from different patient cohorts and datasets. This model can accurately predict the survival status of colon cancer patients at 1, 3, and 5 years, thereby enhancing the model’s versatility. We validated this model using three independent COAD patient cohorts, demonstrating its high stability and accuracy in predicting the survival rates of colon cancer patients. We also developed a nomogram based on the GBM scoring model and various clinical information of colon cancer to facilitate the use of our model.

However, despite achieving some progress in our research, there are still some limitations. Firstly, due to the retrospective design of our study, there is a certain selection bias and information bias. In the future, we will need more prospective clinical datasets to validate our findings. Secondly, our prognostic model is constructed based on gene expression values, yet cancer development is a complex multifactorial process. Apart from genetic variations, factors like epigenetic changes may also influence cancer development, potentially affecting the predictive capability of our model. Thirdly, our model is built on multiple genes, and further research and validation are needed on the specific biological functions of these genes in colon cancer.

In conclusion, our study provides new insights into subtyping colon cancer. This prognostic model not only aids in improving survival prediction for colon cancer patients but may also be applicable in distinguishing subtypes of other types of cancer.

## 4. Materials and Methods

### 4.1. Cell Culture

The human colon cancer cell lines LoVo, SW620, SW480, HCT116, HT-29, and the human normal colon epithelial cell line CCD841 CON were all sourced from ATCC. In the experiments, all cell lines have undergone routine mycoplasma contamination testing, with negative results. These cell lines are cultured in DMEM medium containing 10% fetal bovine serum and 1% penicillin–streptomycin.

### 4.2. Human Colon Cancer Tissue Samples

With the approval of the Ethics Committee of Qingdao University (QDU-HEC-2022046), we collected clinical tumor tissue samples from 22 patients with colon cancer, as well as normal colon tissue samples (Supplementary Table S2). These patients consisted of 10 females (average age 64 years) and 12 males (average age 62 years). All tissue samples were rapidly frozen in liquid nitrogen immediately after the completion of the patients’ surgeries and stored in a −80 °C freezer.

### 4.3. Real-Time PCR (qPCR)

For all colon cancer cell lines and clinical tissue samples, we extracted total RNA using TRIzol reagent. The specific procedure involved homogenizing the samples with TRIzol reagent, adding chloroform for phase separation, dividing the mixture into a transparent upper aqueous phase containing RNA, an interphase, and a pink lower organic phase containing DNA and proteins. Subsequently, RNA was precipitated from the upper aqueous phase using isopropanol. Following the manufacturer’s instructions, we used 1 μg of RNA and reverse transcribed it into cDNA using the HiScript III RT SuperMix kit for qPCR reactions. We then performed qPCR using the QuantStudioTM 3 system and QuantiTect SYBR Green I PCR premix kit, and analyzed the results using QuantStudio 3 Flex software. The reference list for the qPCR primer is provided in Supplementary Table S3.

### 4.4. Source and Pretreatment of Colon Cancer Datasets

This study includes data from three independent colon cancer datasets: the TCGA-COAD dataset (Supplementary Table S4) from The Cancer Genome Atlas (https://portal.gdc.cancer.gov/, TCGA, accessed on 12 March 2024) database, and the GSE17538 and GSE29623 datasets from the Gene Expression Omnibus (http://www.ncbi.nlm.nih.gov/gds, GEO, accessed on 10 March 2024). After analyzing the clinical information from these cohorts, patients with incomplete follow-up time were excluded. Subsequently, we used the R package to convert all expression data in these three datasets to log2(expression + 1) format. Finally, we conducted clustering, signature development, and prognosis model construction using data from the TCGA-COAD dataset, while the remaining three datasets were utilized as validation sets. Our research workflow is illustrated in Supplementary Figure S1.

### 4.5. Unsupervised Clustering of Seven TAS2Rs Prognostic Genes

In this study, we identified seven TAS2Rs genes associated with the prognosis of colon cancer patients by using univariate Cox regression analysis (*p* < 0.05). Subsequently, based on the expression data of these seven TAS2Rs prognostic genes, different molecular subtypes of colon cancer patients were identified using unsupervised clustering methods. The analysis was performed using the R package ConsensuClusterPlus with 1000 repetitions to ensure clustering stability. The partitioning around the medoid (PAM) method and Euclidean distance were utilized to quantify the similarity of gene expression profiles among patients, and the area under the cumulative distribution function (CDF) curve was used to determine the optimal clustering result k. Ultimately, two clusters (k = 2) were identified and named Cluster A and Cluster B.

### 4.6. PD-1/CTLA4 Treatment Response

To predict the response of different subtypes of colon cancer patients to immunotherapy, we downloaded immunotherapy prediction information from the cancer immunome database (https://tcia.at/home, TCIA, accessed on 20 March 2024) (Supplementary Table S5). This database provides comprehensive immune genomic analysis results of 20 solid cancers from TCGA and other data sources using next-generation sequencing (NGS) data. Among them, the immunophenotype score (IPS) can be used to predict the response of cancer patients to immunotherapy drugs targeting PD-1 and CTLA4. By analyzing IPS data, we could assess the expected response of different subtypes of colon cancer patients to immunotherapy drugs, thus providing an important reference for clinical treatment.

### 4.7. Single Sample Gene-Set Enrichment Analysis (ssGSEA)

ssGSEA is a technique that extends the GSEA method and is widely used in bioinformatics research related to immune infiltration [56]. We evaluated the types of immune cells and the degree of immune cell infiltration in the TCGA-COAD expression profile using the R package GSVA. This method takes gene expression data and gene sets as input, ranks the input genes based on their expression levels, and calculates an enrichment score (ES) based on the input data. Gene-set annotations of TME infiltrating immune cell types were obtained from studies by Charoentong et al. [57,58]. This research encompasses various human immune cell subtypes including activated CD8+ T cells, activated dendritic cells, macrophages, natural killer T cells, regulatory T cells, and others. We used one-way ANOVA to test the immune cell infiltration between different subtypes. The ES calculated by ssGSEA analysis represents the relative abundance of each infiltrating cell type in the TME of each sample, helping us understand the immune cell composition and infiltration levels of different colon cancer subtypes.

### 4.8. Identification of DEGs in the Two Subtypes of Colon Cancer

To identify the DEGs between Cluster A and Cluster B subtypes of colon cancer patients, we utilized the R package limma. Limma is a powerful tool for differential expression analysis, employing an empirical Bayesian approach to efficiently estimate changes in gene expression [59]. In this study, we employed the limma package to identify DEGs between Cluster A and Cluster B, where a *p*-value < 0.001 was considered to have significant statistical differences.

### 4.9. Functional Enrichment Analysis of DEGs

Gene ontology (GO) provides information about gene functions, cellular components, and biological processes derived from various biological function information from microarrays and other large datasets [60]. The Kyoto Encyclopedia of Genes and Genomes (KEGG) is a systematic database of gene and genome function information, primarily stored in PATHWAY sections [61]. To further understand the biological functions of DEGs and the signaling pathways involved, we utilized the R package clusterProfiler for GO biological function and KEGG pathway analysis. In this analysis, we conducted statistical tests on the functional annotation and pathway enrichment analysis of DEGs to determine which functions and pathways are significantly associated with differentially expressed genes, with *p* < 0.05 considered statistically significant.

### 4.10. Gene-Set Enrichment Analysis (GSVA)

GSVA is a nonparametric, unsupervised computational method used to calculate the enrichment score of specific gene sets in each sample, typically employed to explore signaling pathways and biological processes within expression data samples [62]. Utilizing the R package GSVA, we conducted an analysis on the enrichment levels of biological mechanisms and KEGG pathways in different subtypes of colon cancer patients. We downloaded the gene set “c2.cp.kegg.v7.2.symbols.gmt” from the MsigDB (https://www.gsea-msigdb.org/gsea/msigdb, accessed on 26 March 2024) dataset for GSVA analysis, evaluating the enrichment levels of these KEGG pathways across the different subtypes of colon cancer. In this analysis, a significance level of *p* < 0.05 was considered statistically significant. To elucidate the differential signaling pathways between the two subtypes of colon cancer, we selected the intersection pathways of Cluster A and Cluster B, visualizing the enrichment levels of these pathways in the form of a heatmap.

### 4.11. Construction of Prognostic Models

We randomly divided the TCGA-COAD dataset into training and testing sets in a 1:1 ratio. To address batch effects between different data, we utilized 381 prognosis-related DEGs (Supplementary Table S6) for gene pairing. Previous studies have validated the robustness of this gene-pairing method, which effectively reduces batch effects without altering the original data distribution, applicable to RNAseq and microarray data [63]. The specific procedure involves labeling the feature “A|B” as 1 if gene A > gene B in expression level, otherwise 0, as shown below:

Characteristic: “Gene A|Gene B”
= {1, Expression(A) > Expression(B);
0, Expression(A) ≤ Expression(B)}

Furthermore, if the expression level of gene A is higher than gene B in all samples, the feature gene pair A|gene B in all samples is marked as 1. These features do not contain classification information as they only consist of “0” and “1” information. Therefore, gene pairs with label frequencies <0.2 or >0.8 were removed from the training set. Using gene-pairing methods, over 18,000 feature gene pairs were identified and further simplified to 30 gene pairs through univariate Cox regression (*p* < 0.05) and LASSO regression. Subsequently, 16 gene pairs (Supplementary Table S7) related to survival were identified through multivariate Cox regression analysis. Finally, a scoring model containing 16 gene pairs was constructed using the gradient boosting machine (GBM) method. Throughout the process, the glmnet, survival, and survminer packages in the R language were utilized.

### 4.12. Validation of Prognostic Models

To evaluate the prognostic performance of gene-pair scoring models, we conducted Kaplan–Meier survival analysis based on gene-pair model features in the training set of TCGA-COAD and three validation sets. Statistical validation was performed using the log-rank test, with *p* < 0.05 considered statistically significant. Subsequently, we calculated the area under the receiver operating characteristic curve (AUC) to predict overall survival (OS). The predictive performance of the model at different time points was assessed using AUC values for patients at 1, 3, and 5 years.

### 4.13. Construction and Validation of Nomograms

To assess the prognostic performance of gene-pair scoring models, we conducted Kaplan–Meier survival analysis based on features of the gene-pair scoring model in the TCGA-COAD training set and three validation sets. Statistical validation was performed using the log-rank test, where *p* < 0.05 was considered statistically significant. We then calculated the area under the receiver operating characteristic (ROC) curve to predict OS. We evaluated the predictive performance of the model at different time points using the AUC values for patients at 1, 3, and 5 years.

## Data Availability

Publicly available datasets were analyzed in this study. These data can be found at https://portal.gdc.cancer.gov/ (accessed on 12 March 2024), http://www.ncbi.nlm.nih.gov/gds (accessed on 10 March 2024), https://tcia.at/home, TCIA (accessed on 20 March 2024), and https://www.gsea-msigdb.org/gsea/msigdb (accessed on 26 March 2024).

## Supplementary Materials

The following supporting information can be downloaded at: https://www.mdpi.com/article/10.3390/ijms25136849/s1.
