# Supplementary material for: Comprehensive Analysis of the Function and Prognostic Value of TAS2Rs Family-Related Genes in Colon Cancer
Source: Int J Mol Sci. 2024 Jun 21;25(13):6849. doi: 10.3390/ijms25136849 (PMC11241446; doi:10.3390/ijms25136849)
Supplement: Supplementary file 1 [file ijms-25-06849-s001.zip › Supplementary Figure.pdf]

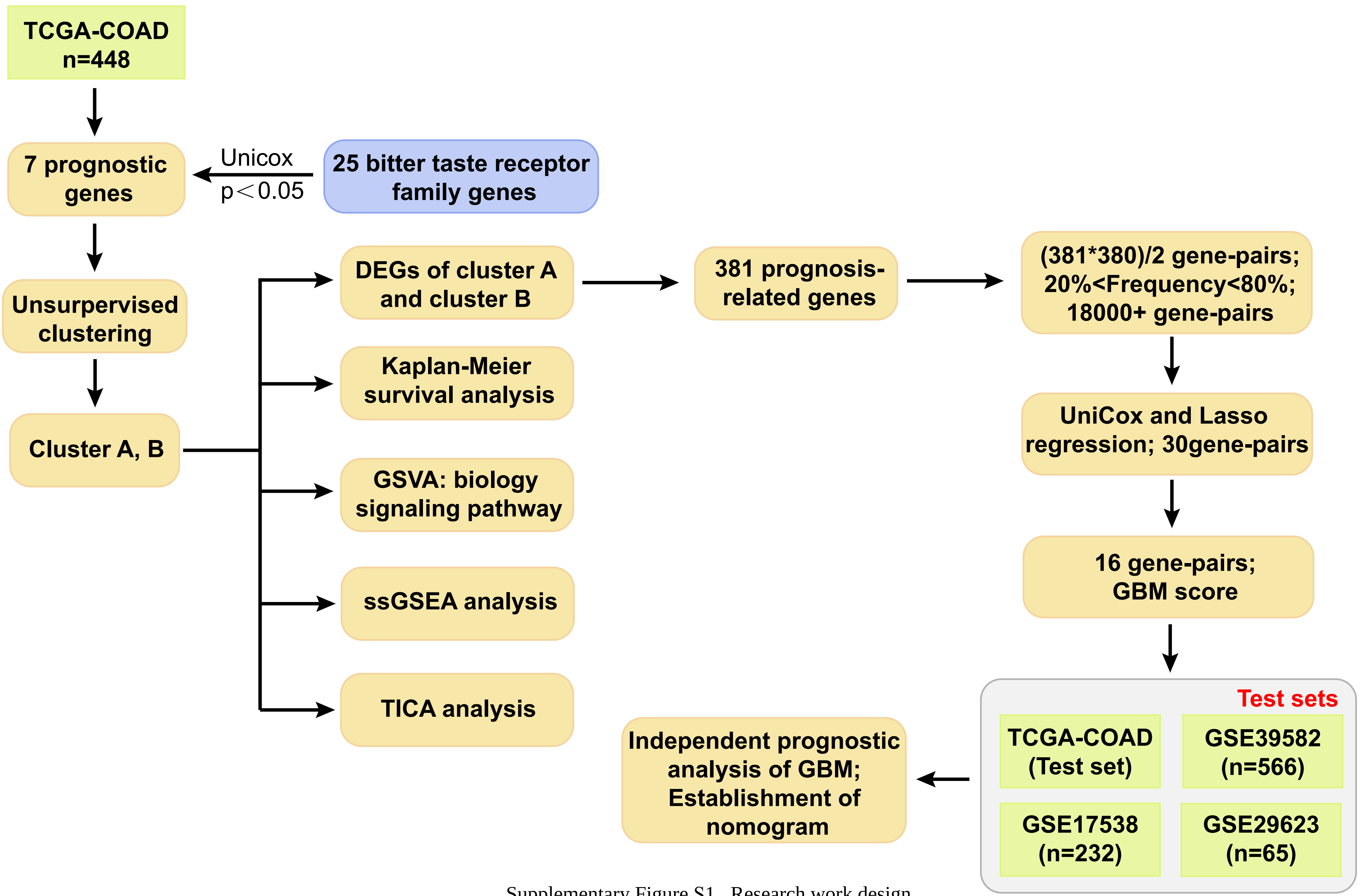

Supplementary Figure S1. Research work design.

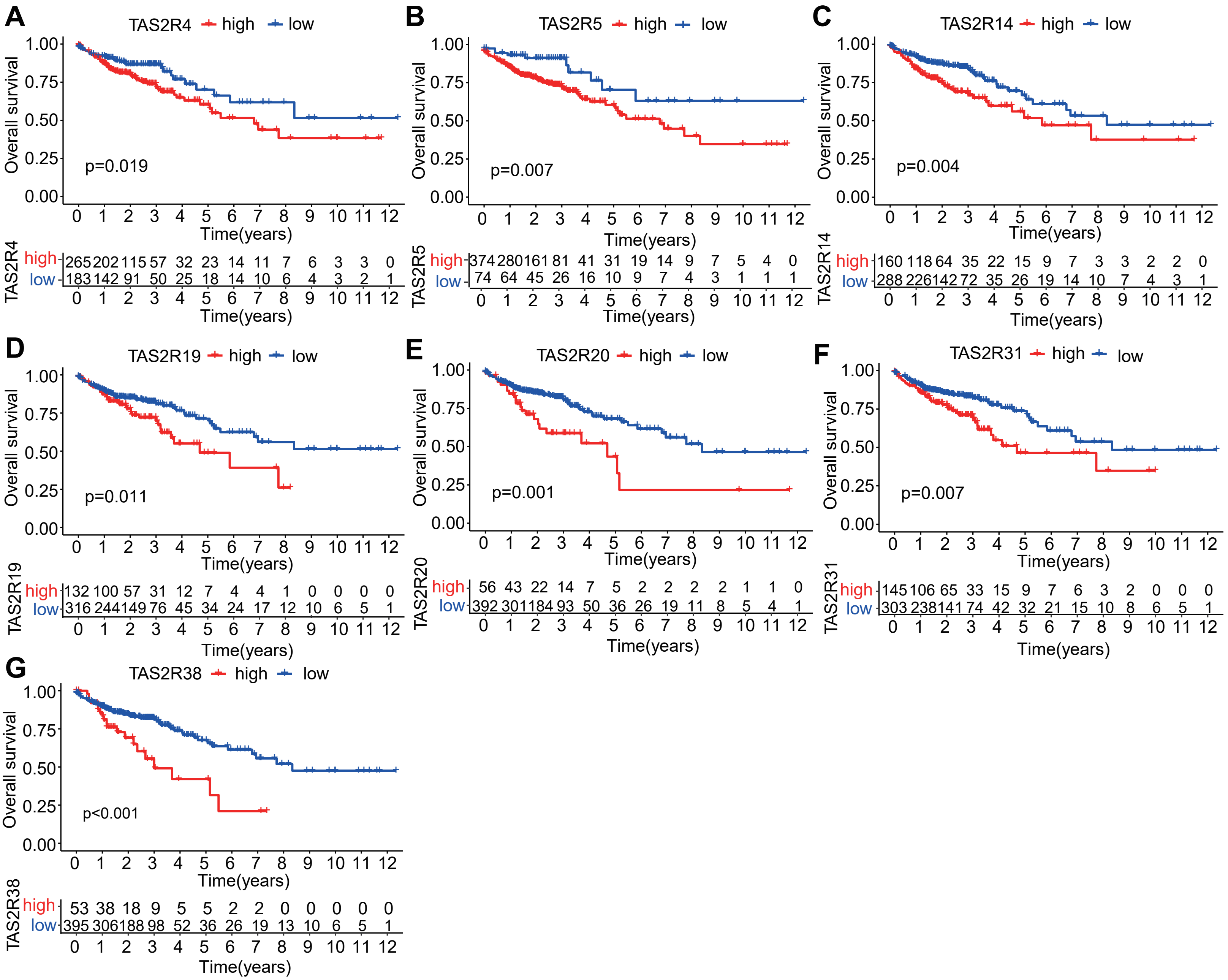

Supplementary Figure S2
Prognostic survival curves of 7 TAS2Rs genes in colon cancer. (A) Survival curves of patients with high and low expression levels of TAS2R4 in TCGA-COAD. (B) Survival curves of patients with high and low expression levels of TAS2R5 in TCGA-COAD. (C) Survival curves of patients with high and low expression levels of TAS2R14 in TCGA-COAD. (D) Survival curves of patients with high and low expression levels of TAS2R19 in TCGA-COAD. (E) Survival curves of patients with high and low expression levels of TAS2R20 in TCGA-COAD. (F) Survival curves of patients with high and low expression levels of TAS2R31 in TCGA-COAD. (G) Survival curves of patients with high and low expression levels of TAS2R38 in TCGA-COAD. Red indicates high gene expression; blue indicates low gene expression (Log-rank test, $p < 0.05$).

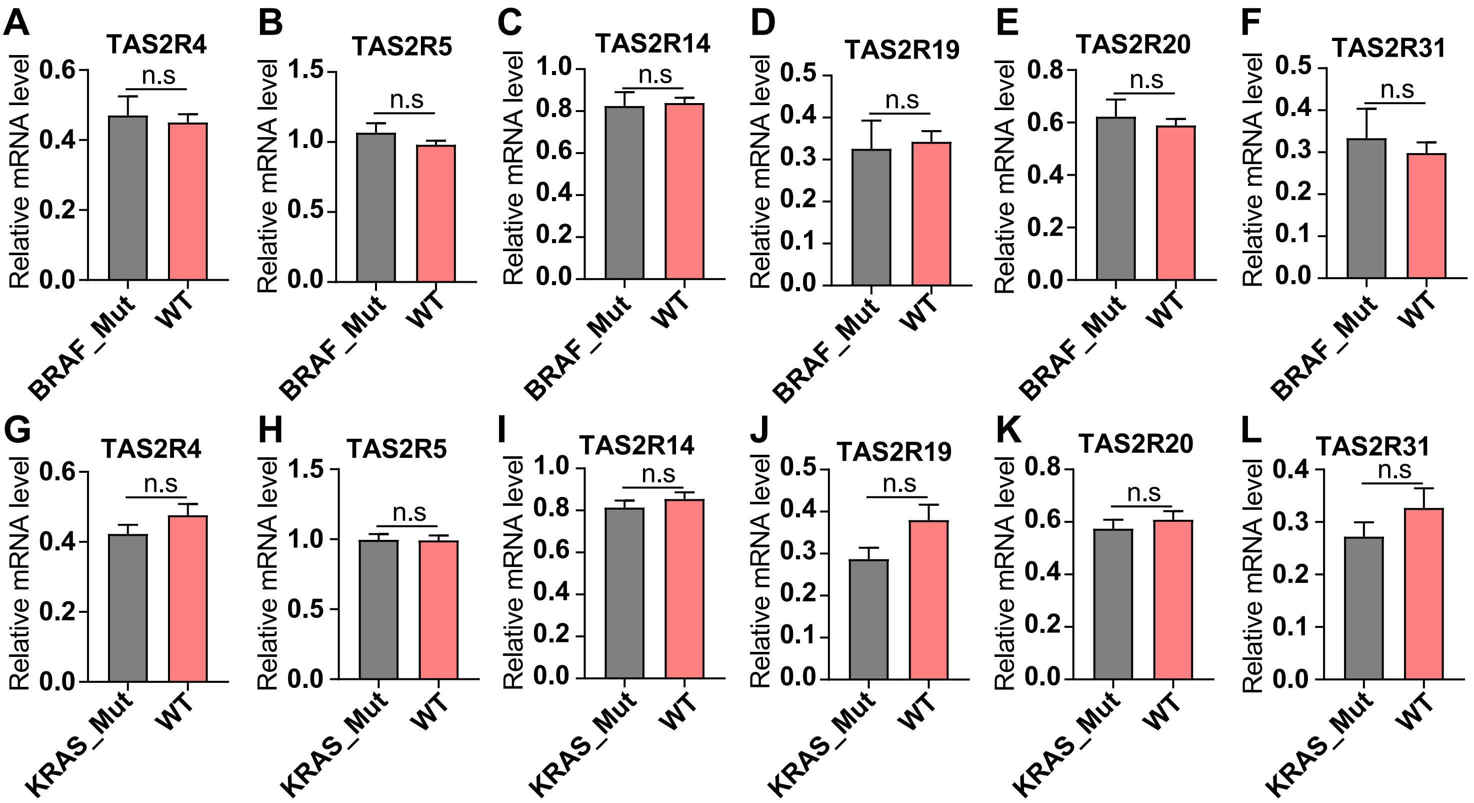

Supplementary Figure S3
(A-F) Expression of TAS2Rs gene in patients with BRAF mutation and wild-type colon cancer. (G-L) Expression of TAS2Rs gene in patients with KRAS mutation and wild-type colon cancer

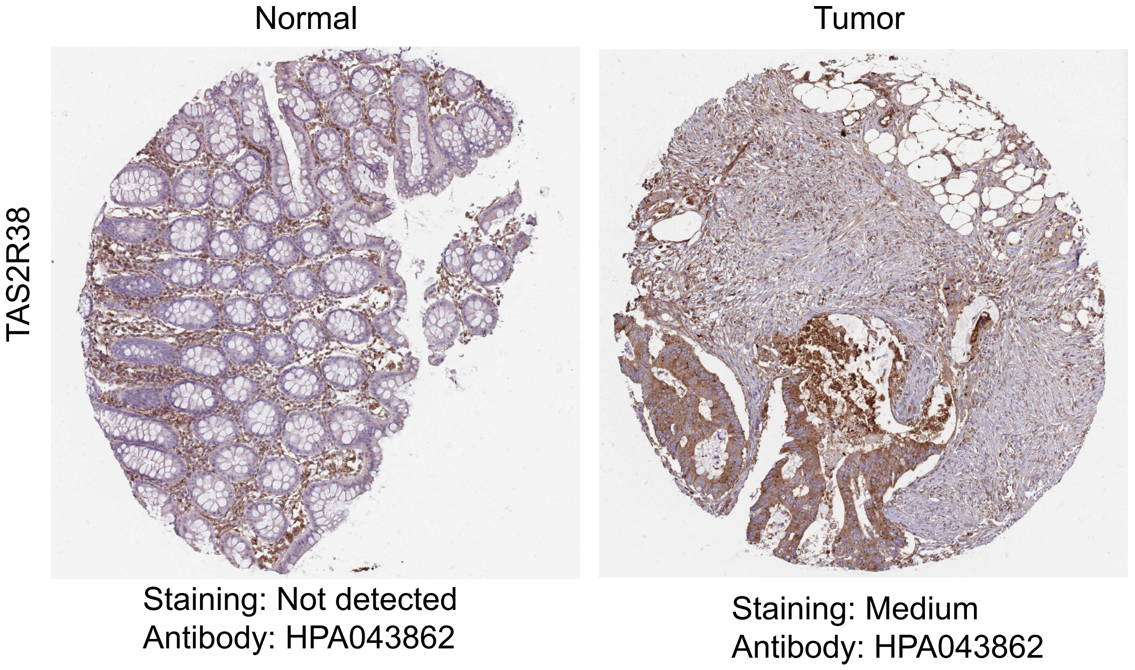

Supplementary Figure S4 Validation of TAS2R38 at the protein level.

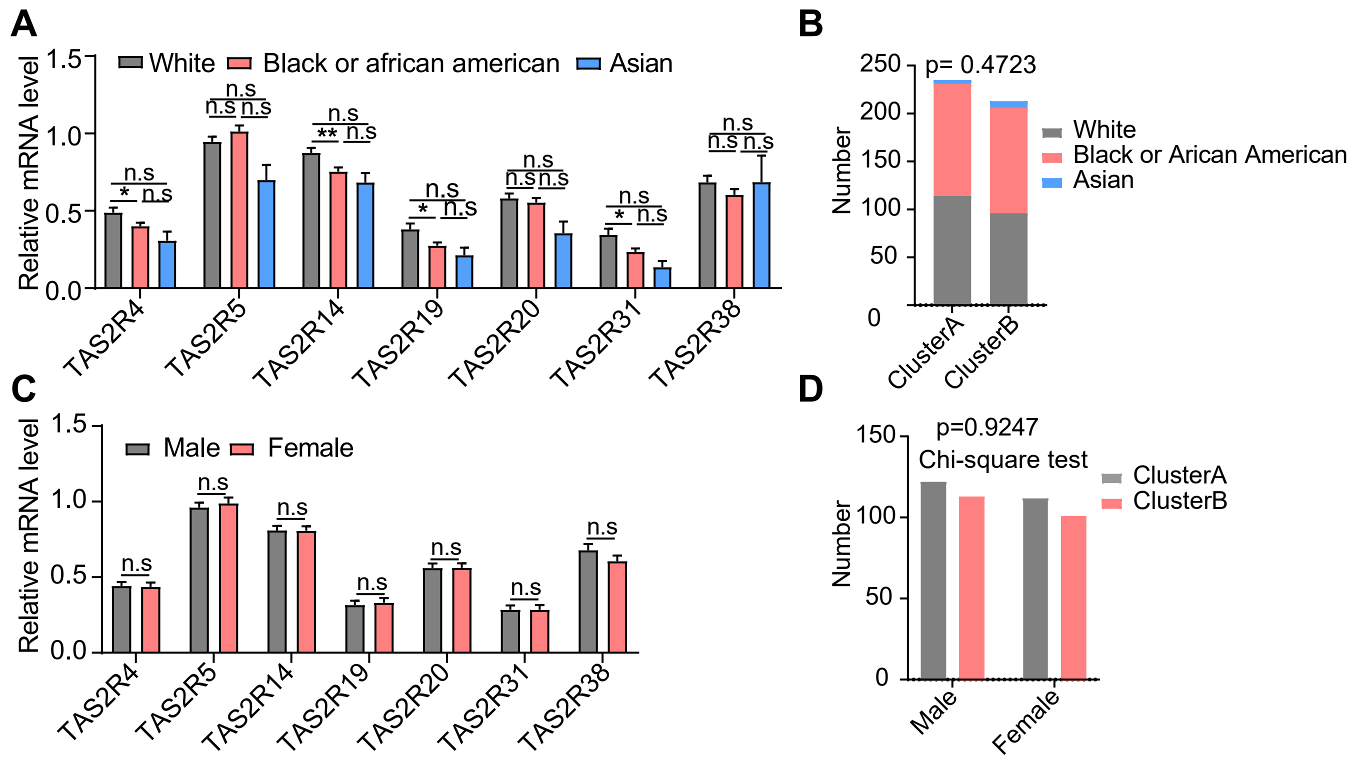

Supplementary Figure S5

Expression characteristics of 7 TAS2Rs in different clinical features. (A) Expression levels of 7 TAS2Rs in different races. (B) Number of different races in Cluster A and Cluster B (Chi-square test). (C) Expression levels of 7 TAS2Rs in different sex. (D) Number of persons of different sex in Cluster A and Cluster B (Chi-square test).

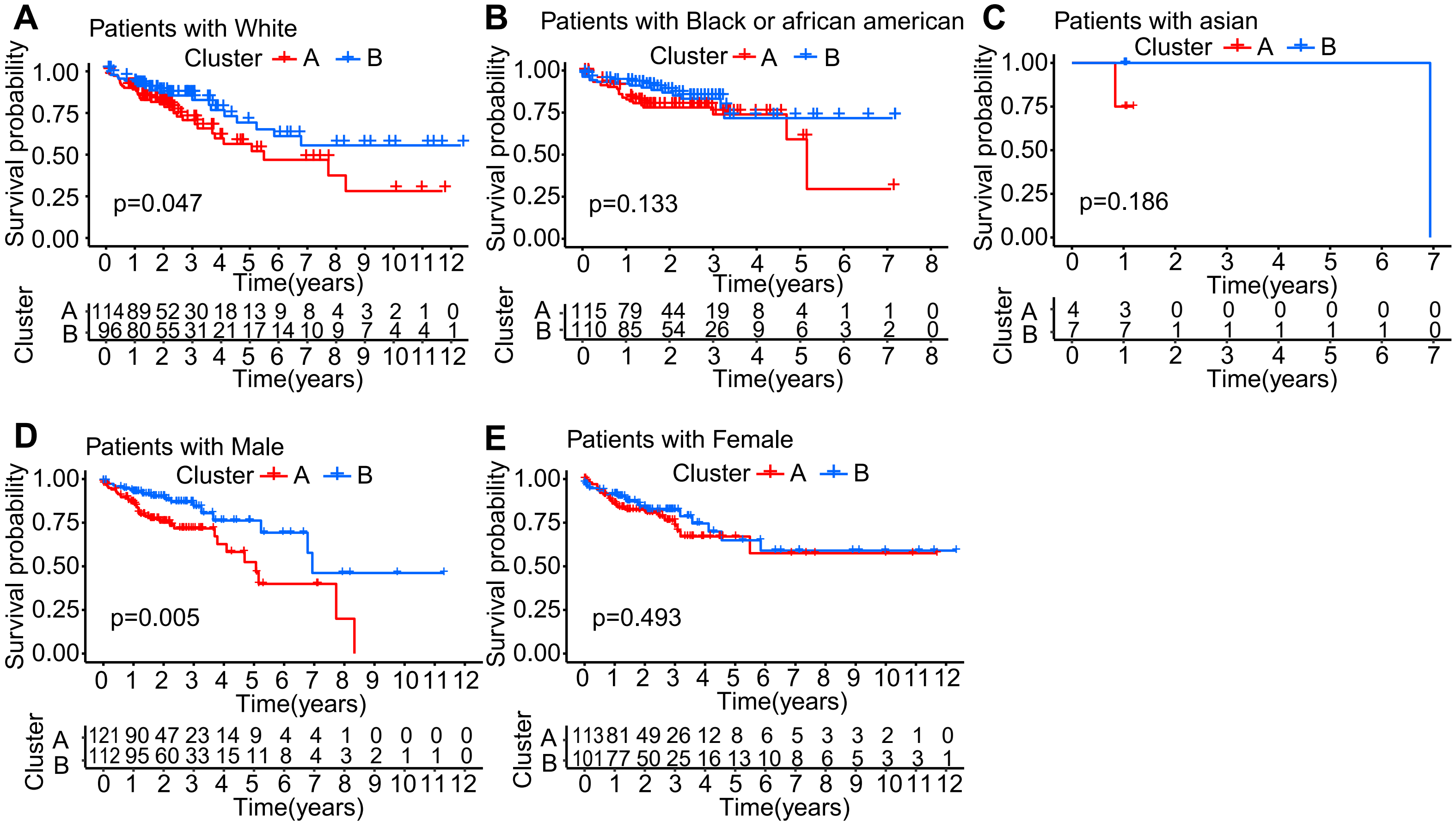

Supplementary Figure S6

Survival curves of two subtypes of colon cancer in different clinical features. (A) Survival curves for subtypes Cluster A and Cluster B of colon cancer in the TCGA dataset of White. (B) Survival curves for subtypes Cluster A and Cluster B of colon cancer in the TCGA dataset of Black or African American. (C) Survival curves for subtypes Cluster A and Cluster B of colon cancer in the TCGA dataset of Asian. (D) Survival curves for subtypes Cluster A and Cluster B of colon cancer in the TCGA dataset of Male. (F) Survival curves for subtypes Cluster A and Cluster B of colon cancer in the TCGA dataset of Female.

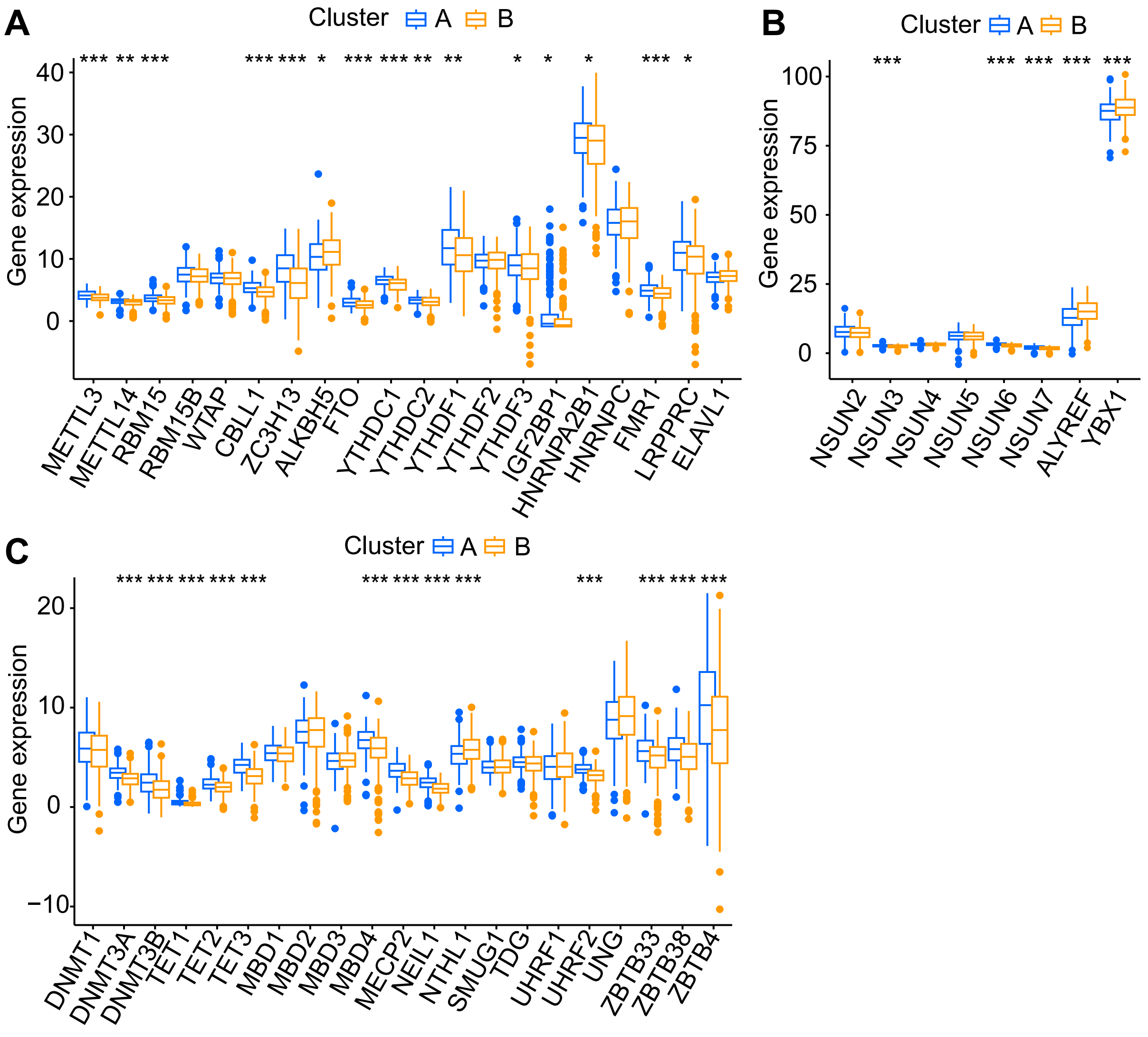

Supplementary Figure S7

Expression of methylation-related genes in the TCGA dataset. (A) Expression of m6A methylation-regulated genes in the TCGA dataset. (B) Expression of m5C methylation-regulated genes in the TCGA dataset. (A) Expression of 5mC methylation-regulated genes in the TCGA dataset.

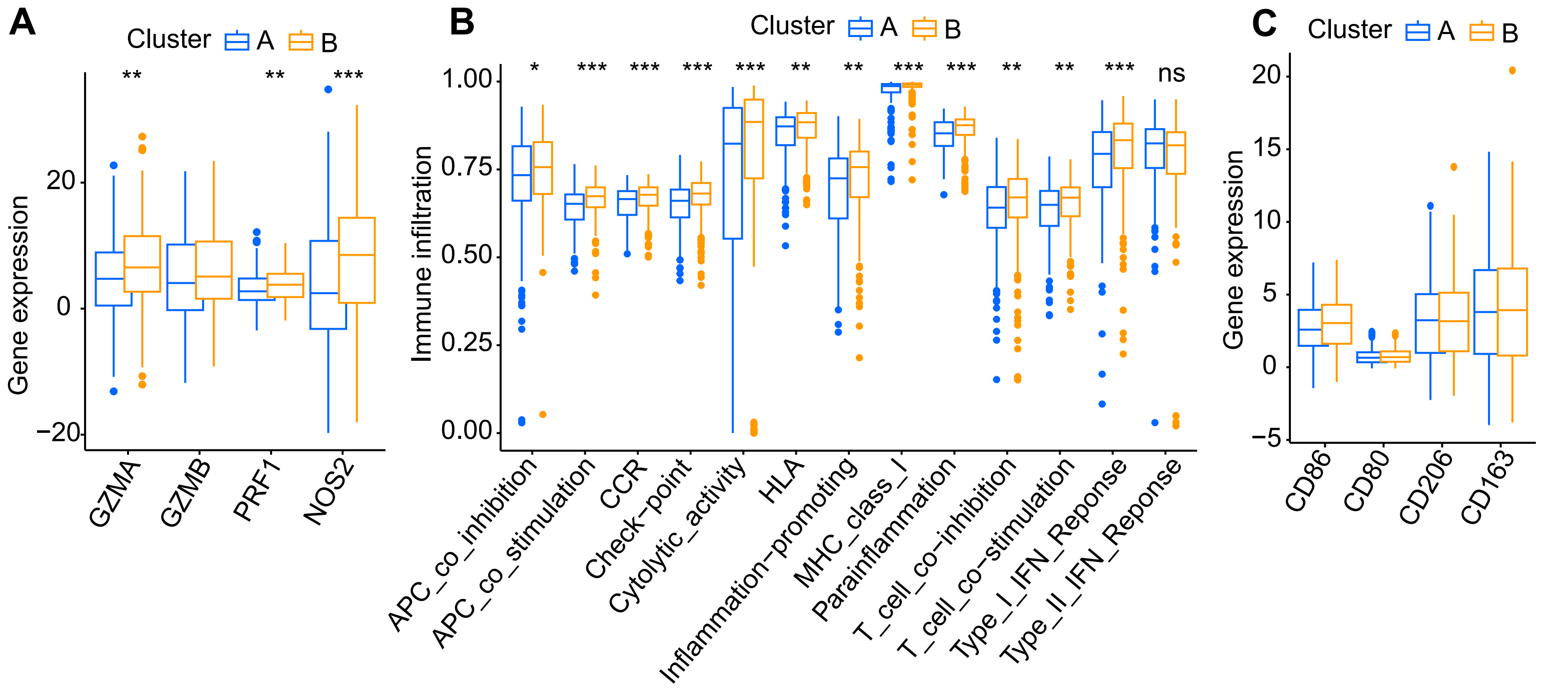

Supplementary Figure S8
Immune cell infiltration characteristics of two molecular subtypes of colon cancer. (A) Expression of CD8+ and CD4+ effector molecules between two subtypes of colon cancer. (B) Analysis of immune-related processes between two subtypes of colon cancer using ssGSEA. (C) Expression of M1 and M2 macrophage markers in two molecular subtypes of colon cancer.

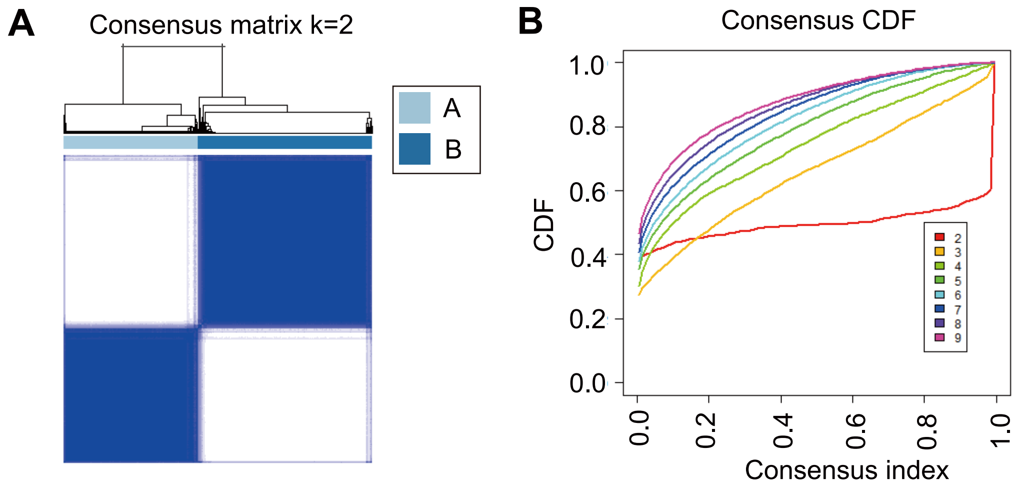

Supplementary Figure S9
Construction of colon cancer genomic subtypes. (A) Consensus matrix of TCGA -COAD for k=2. (B) CDF curves in consensus clustering analysis. CDF curves representing consensus scores for different subtype numbers (k=2-9).

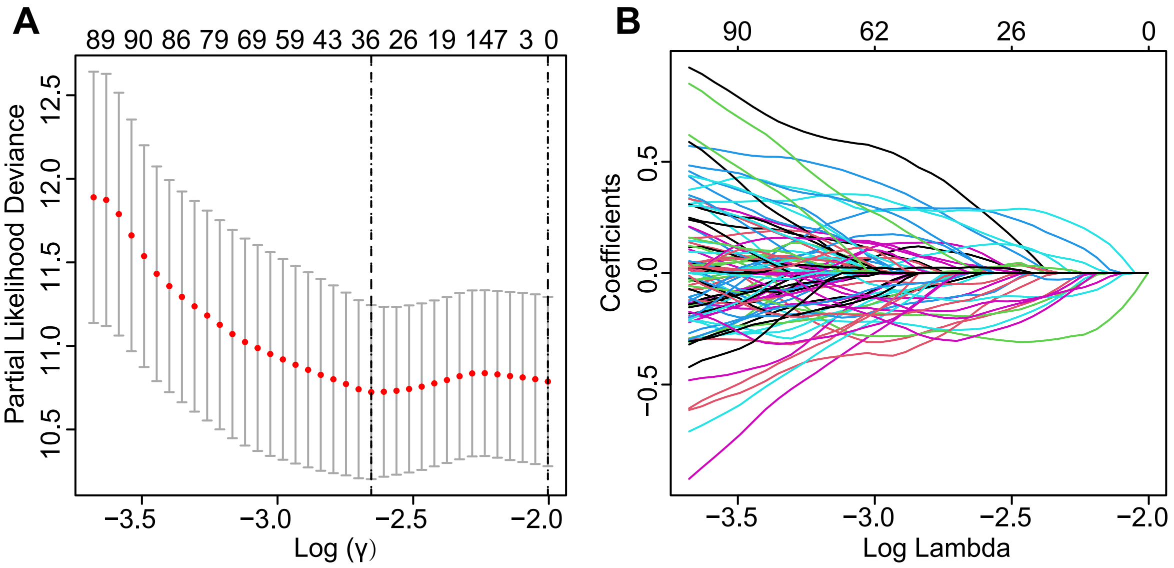

Supplementary Figure S10 Lasso regression analysis diagram.

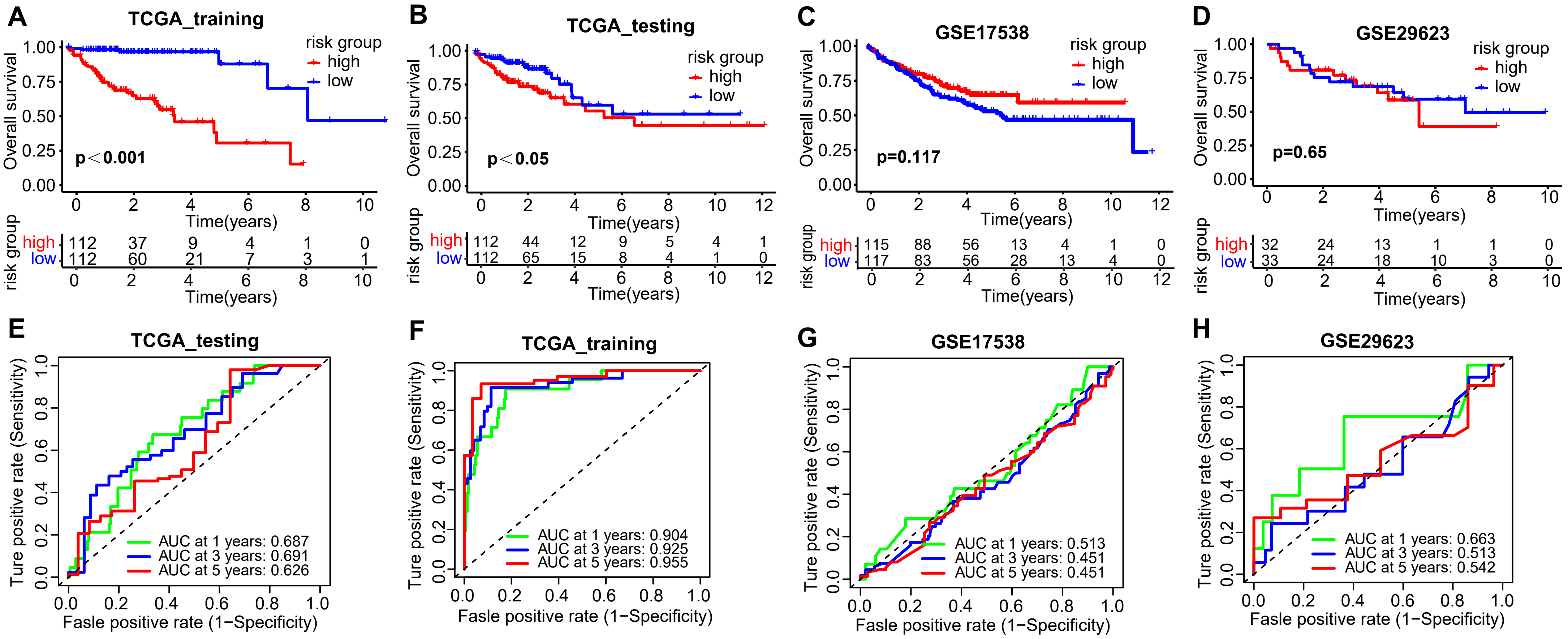

Supplementary Figure S11

Construction of a scoring model based on the Random Forest (RF) algorithm. (A-D) Survival curves of the RF scoring model in TCGA-COAD training set, TCGA-COAD test set, GSE17538, GSE29623 (Log-rank test). (E-H) ROC curves for 1 year, 3 years, and 5 years RF scoring models in TCGA-COAD training set, TCGA-COAD test set, GSE17538, GSE29623.

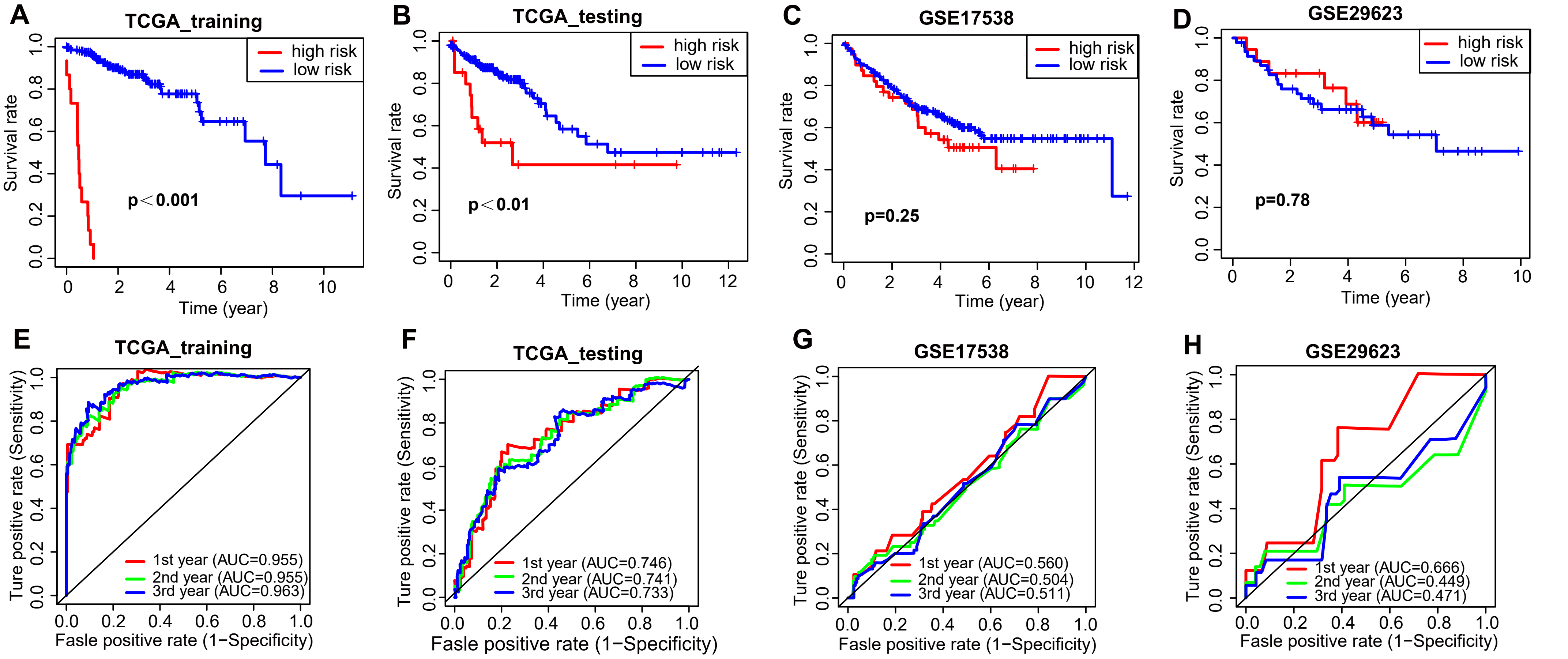

Supplementary Figure S12

Construction of a scoring model based on Cox regression analysis. (A-D) Survival curves of Cox regression analysis scoring model in TCGA-COAD training set, TCGA-COAD test set, GSE17538, GSE29623 (Log-rank test). (E-H) ROC curves of Cox regression analysis models at 1 year, 3 years, and 5 years in TCGA-COAD training set, TCGA-COAD test set, GSE17538, GSE29623.

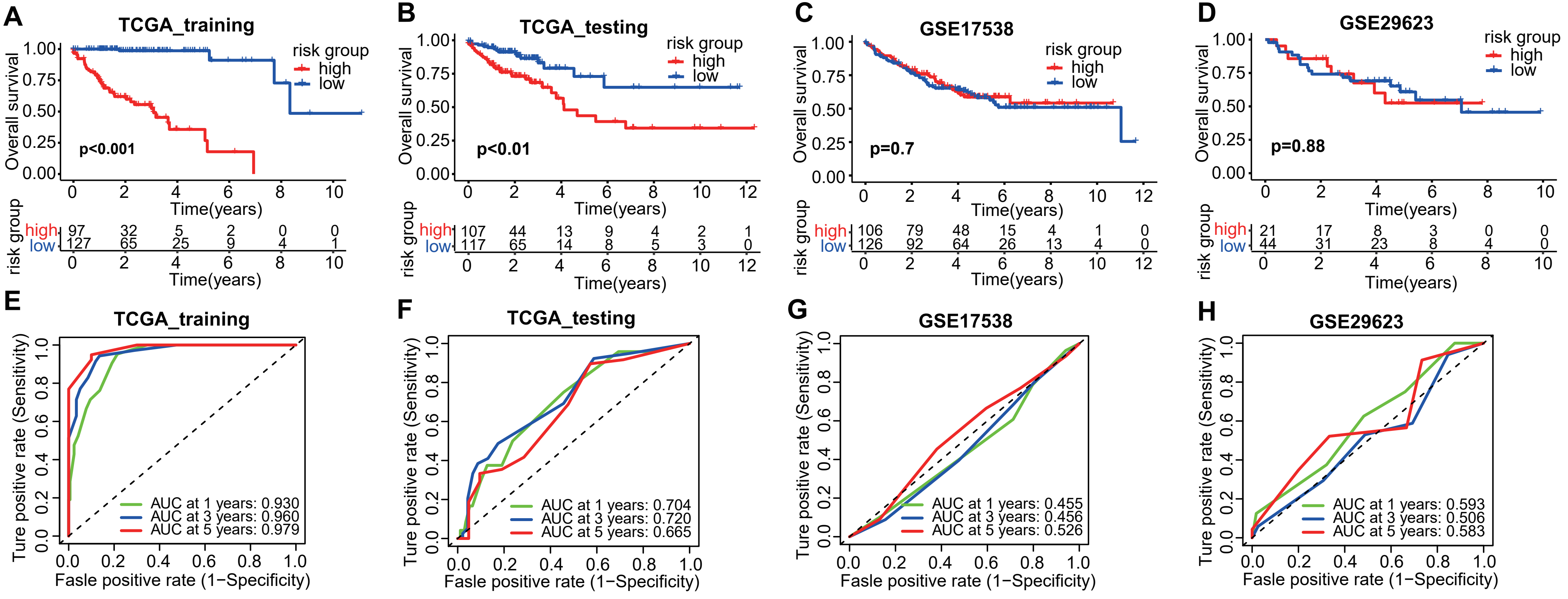

Supplementary Figure S13
Construction of a scoring model based on the Conditional Inference Forest (CIF) algorithm. (A-D) Survival curves of the CIF scoring model in TCGA-COAD training set, TCGA-COAD test set, GSE17538, GSE29623 (Log-rank test). (E-H) ROC curves for 1 year, 3 years, and 5 years CIF scoring models in TCGA-COAD training set, TCGA-COAD test set, GSE17538, GSE29623.

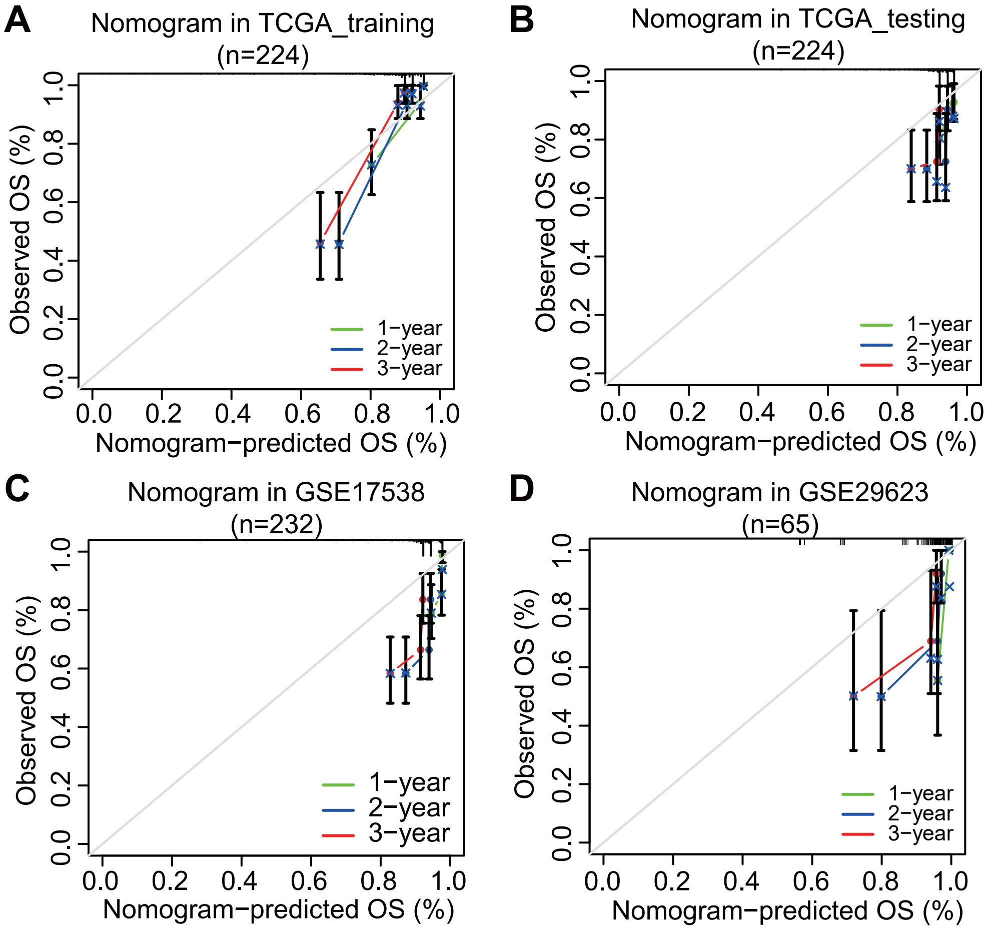

Supplementary Figure S14

Calibration curve of the nomogram. (A) Calibration curve of the nomogram in the TCGA-COAD training set. (B) Calibration curve of the nomogram in the TCGA-COAD test set. (C) Calibration curve of the nomogram in the GSE17538. (D) Calibration curve of the nomogram in the GSE29623.

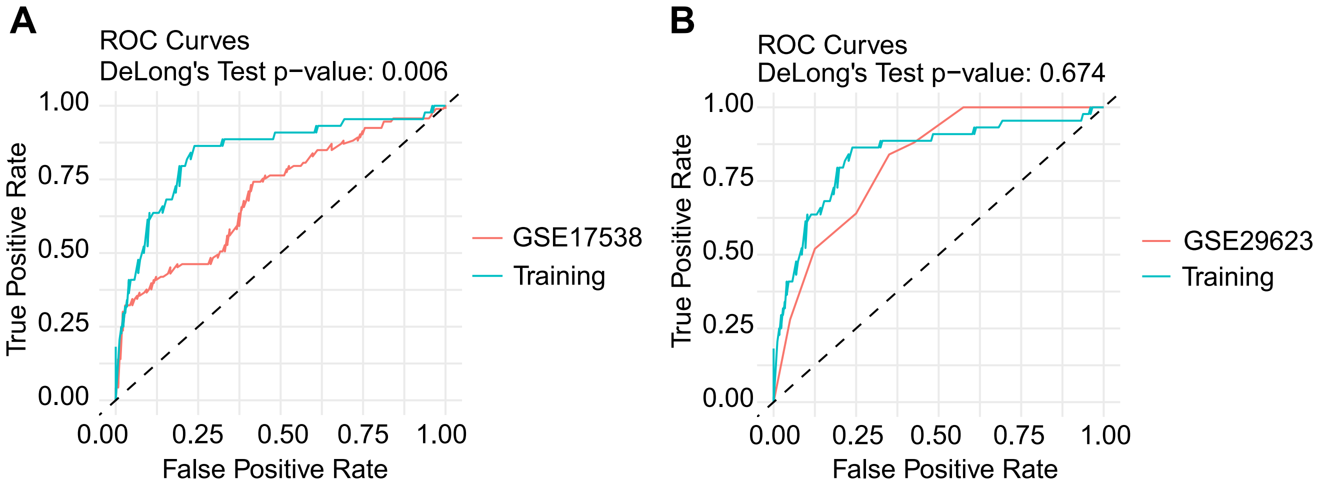

Supplementary Figure S15
(A) AUC-ROC values for the training set and the external validation set GSE17538. (B) AUC-ROC values for the training set and the external validation set GSE29623.
